# Supplementary material for: Histone H3K23-specific acetylation by MORF is coupled to H3K14 acylation
Source: Nat Commun. 2019 Oct 17;10:4724. doi: 10.1038/s41467-019-12551-5 (PMC6797804; doi:10.1038/s41467-019-12551-5)
Supplement: Supplementary file 2 — Supplementary Information [file 41467_2019_12551_MOESM2_ESM.pdf]

Supplementary Information

Histone H3K23-specific acetylation by MORF is coupled to H3K14 acylation

Brianna J. Klein, Suk Min Jang, et al.,

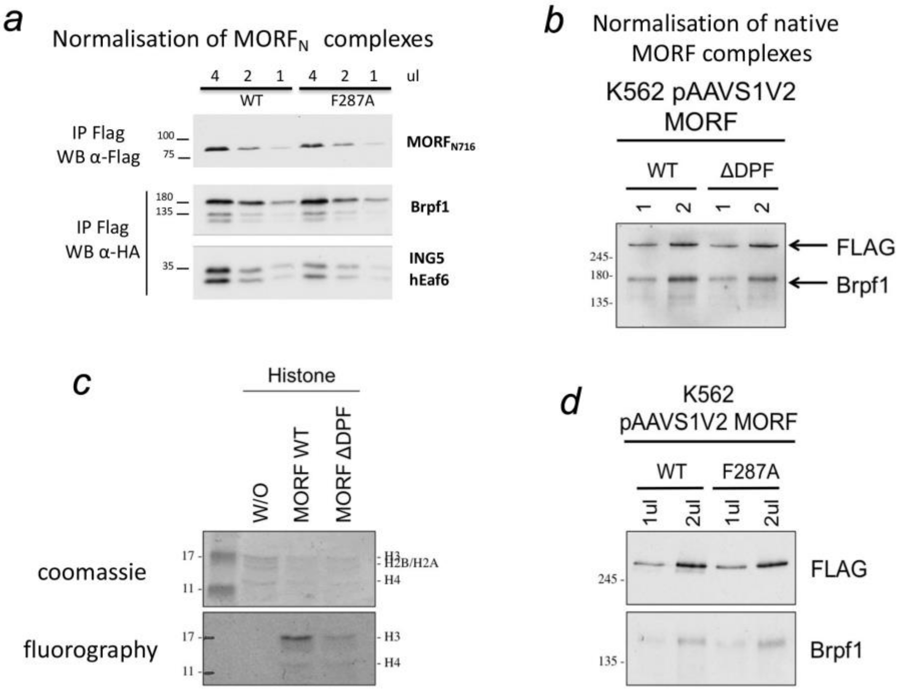

Supplementary Figure 1. MORF complexes used in HAT assays. (a) MORF complexes purified from HEK293T cells after transient co-transfection of Flag-MORF with HA-BRPF1, HA-ING5 and HA-hEaf6. Western blot analysis of flag peptide elution of the 4 subunits and their normalization for HAT assays. (b) Normalization of wild-type and delta-DPF native MORF complexes purified from K562 cells, as evaluated by western blot using Flag and Brpf1 Abs. (c) In-gel HAT assays corresponding to the liquid HAT assay with native wild-type and DPF-deleted MORF complexes on free core histones. The deletion of MORF DPF greatly affects its activity toward free histone H3. (d) Normalization of wild-type and F287A native MORF complexes purified from K562 cells, as evaluated by western blot using FLAG and BRPF1 antibodies. Related to Figures 1 and 4.

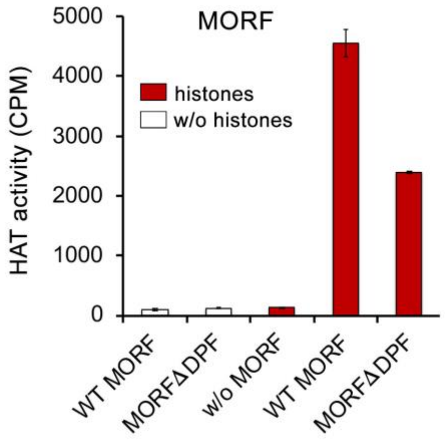

Supplementary Figure 2. The MORF complex is a H3K23-specific HAT. HAT activity of the native MORF WT and MORF ΔDPF complexes from K562 cells on native free core histones. Error bars indicate the range from duplicate samples. Related to Figure 1.

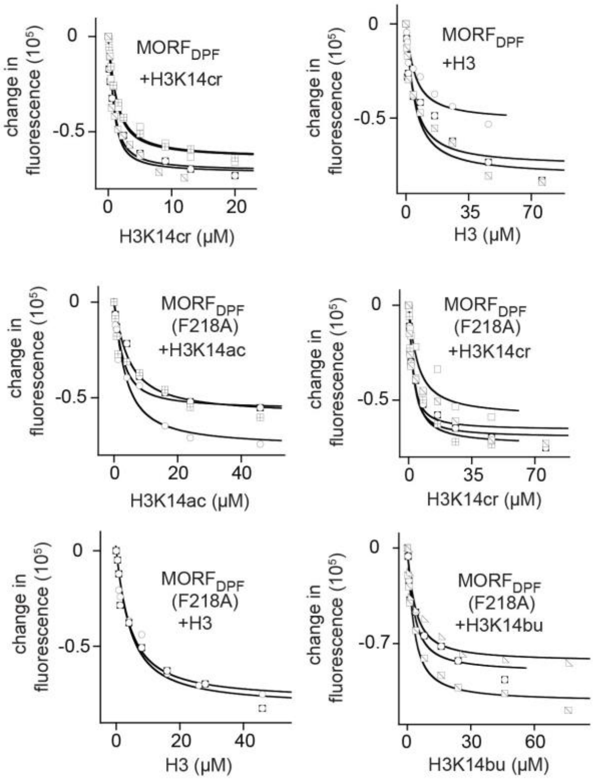

Supplementary Figure 3. Measurements of binding affinities by fluorescence spectroscopy. Binding curves used to determine the K_d values by tryptophan fluorescence for the interactions of WT and mutated MORF$_{DPF}$ to indicated H3 peptides. Related to Figure 2.

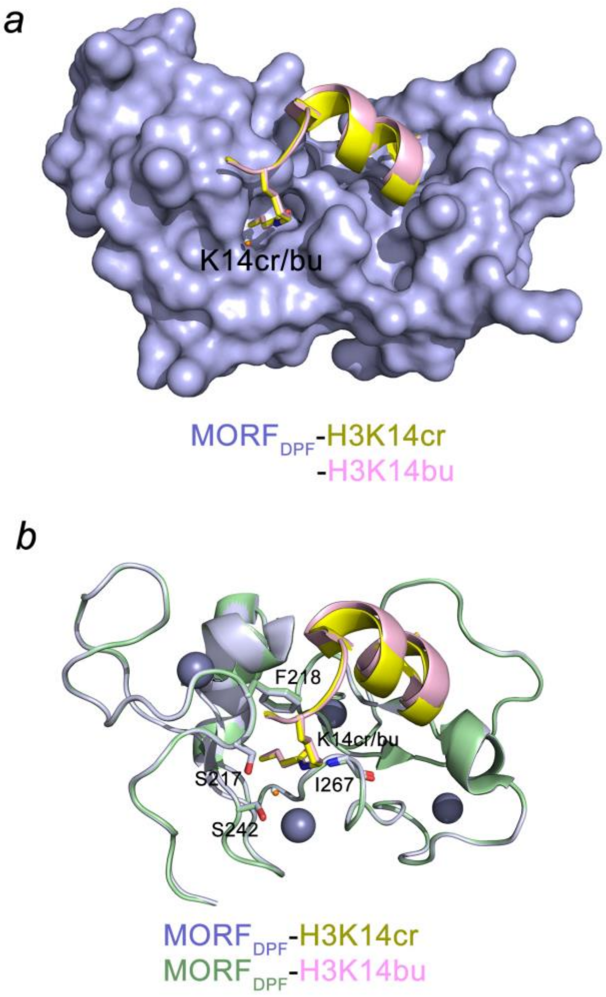

Supplementary Figure 4. The acyllysine-binding mechanism. Structural overlay of $MORF_{DPF}$ in complex with H3K14cr peptide (dark yellow) and H3K14bu peptide (pink) using surface representation (a) or ribbon diagram (b). Related to Figure 3.

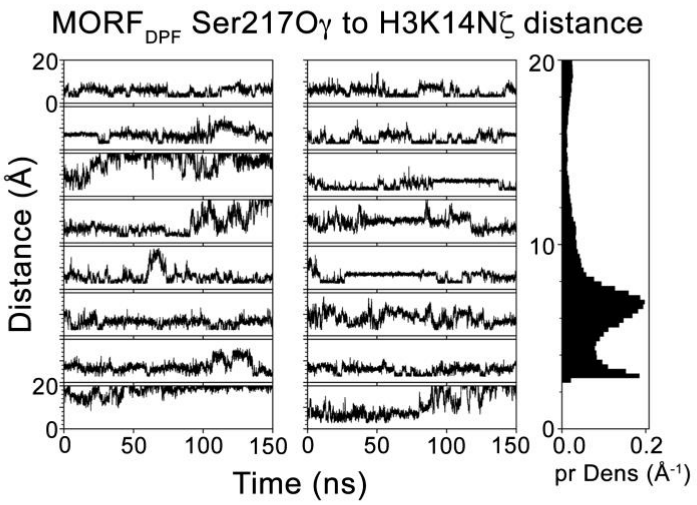

Supplementary Figure 5. MD simulations of the MORF$_{DPF}$-H3 complex. Time-course and distribution of the distance between Ser217Oγ of MORF$_{DPF}$ and K14Nζ of H3 in MD. Related to Figure 3.

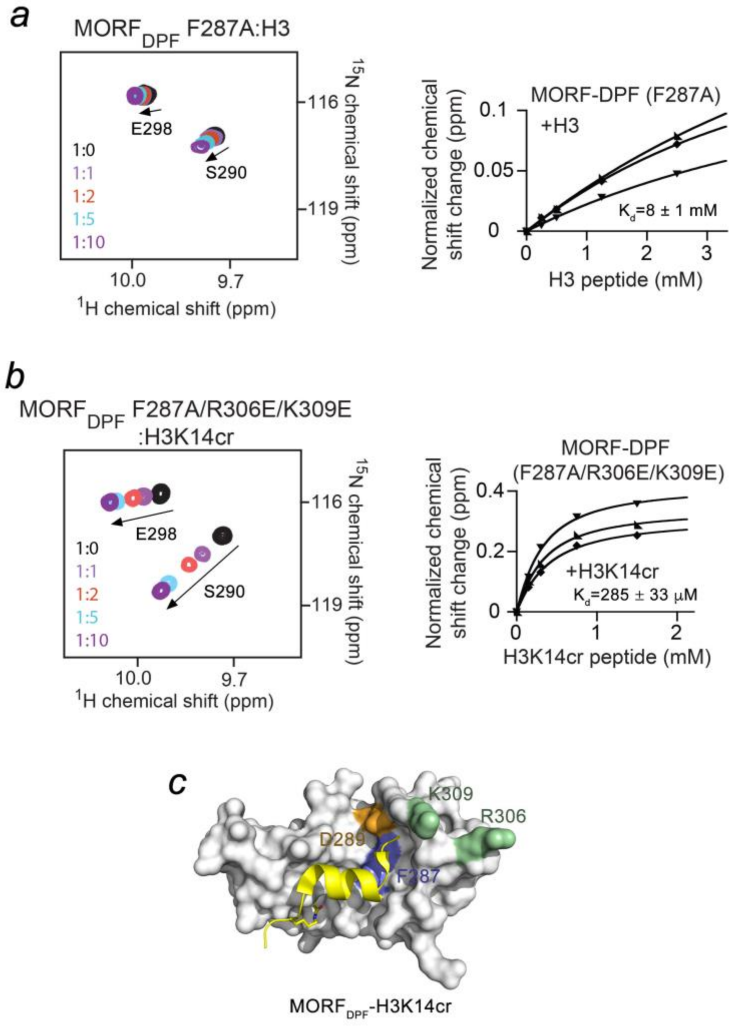

Supplementary Figure 6. The closely positioned histone and DNA binding sites of MORF$_{DPF}$ (a, b) Superimposed ^{1}H,^{15}N HSQC spectra of MORF$_{DPF}$ mutants collected as indicated peptides were added stepwise (left panels). Spectra are color coded according to the protein:peptide molar ratio (inset). Representative binding curves used to determine K_ds for the interactions of MORF$_{DPF}$ mutants with indicated peptides by NMR (right panels). Error bars represent SD. (c) Structure of the MORF$_{DPF}$-H3K14cr complex with mutated residues colored and labeled. Related to Figures 4 and 5.

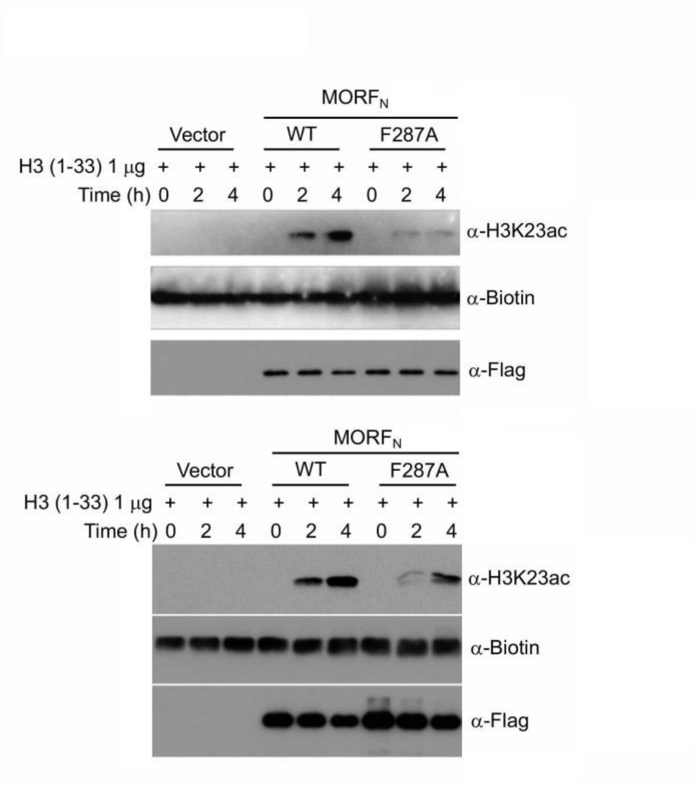

Supplementary Figure 7. Two biological repeats show that point mutation of DPF attenuates HAT activity of MORF *in vitro*. Western blot analysis of HAT assays of purified wild type and mutant MORF$_N$ using H3 (1-33) peptide as substrate. Biotinylated peptides are shown as loading controls. Related to Figure 4.

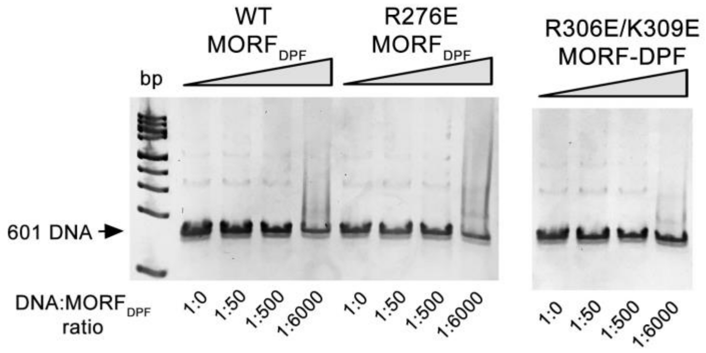

Supplementary Figure 8. The region of MORF$_{DPF}$ containing R306 and K309 is involved in the interaction with DNA. EMSA of 147-bp 601 DNA incubated with increasing amounts of WT or mutated MORF$_{DPF}$. DNA to protein molar ratio is shown below the gel images. Related to Figure 5.

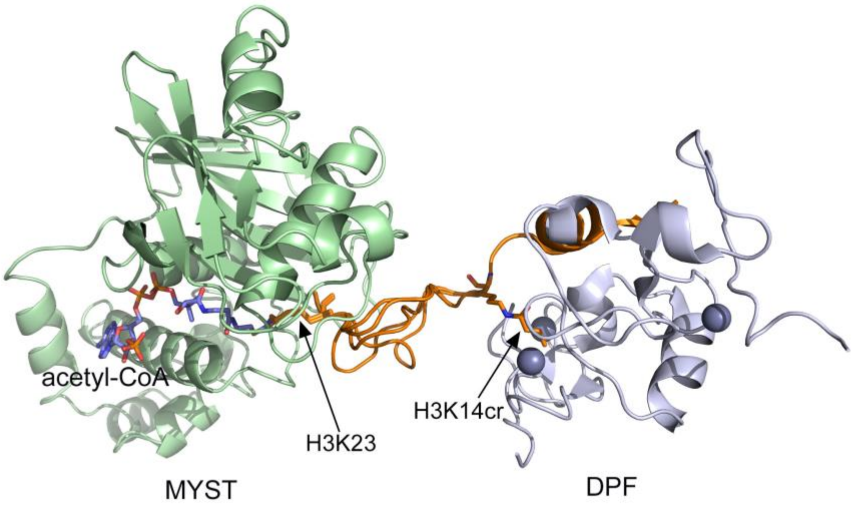

Supplementary Figure 9. The bivalent interaction of the MYST and DPF domains with histone H3 tail. A model showing the interaction of $MORF_{DPF\text{-}MYST}$ with a single H3 tail *in cis*. Related to Figure 8.

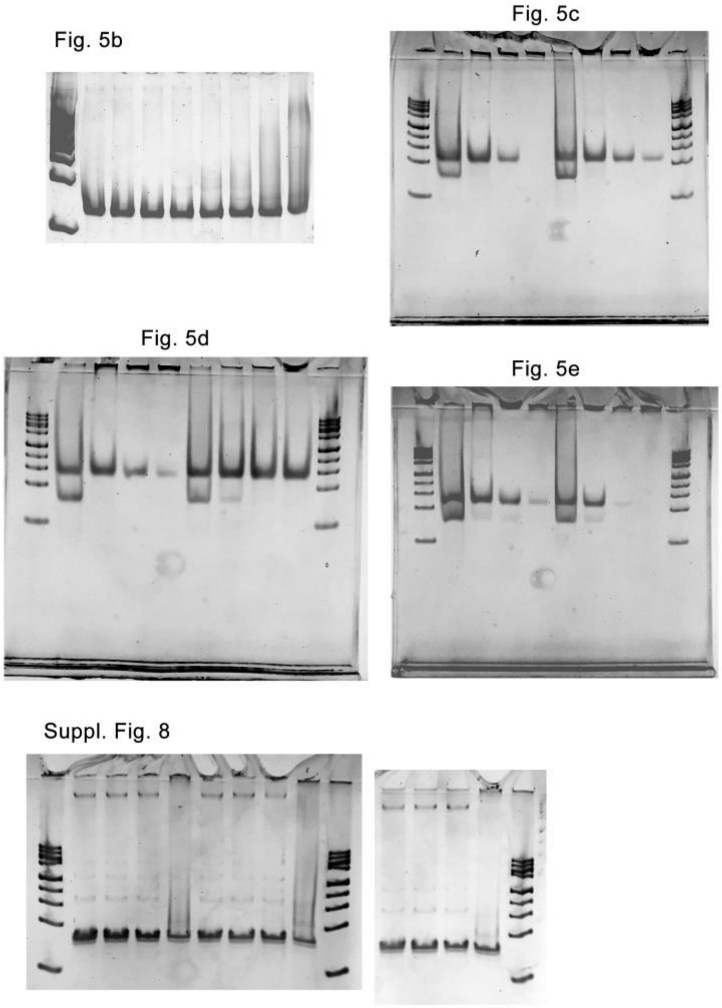

Supplementary Figure 10. Uncropped gels. Related to Figure 5b-e and Supplementary Figure 8.

Supplementary Table 1. Data collection and refinement statistics for the structure of the MORF$_{DPF}$-H3K14cr complex (molecular replacement)

	MORF-DPF:H3K14cr
**Data collection**	
Space group	P 1 21 1
Cell dimensions	
a, *b*, *c* (Å)	32.6, 69.5, 70.2
α, β, γ (°)	90.0, 97.3, 90.0
Resolution (Å)	2.08(2.12-2.08) *
R_{sym} or R_{merge}	9.0(26.4)
I / σI	20.3(6.6)
Completeness (%)	99.9(100.0)
Redundancy	6.3(5.9)
**Refinement**	
Resolution (Å)	24.6-2.08
No. reflections	18589
R_{work} / R_{free}	0.1659/0.2191
No. atoms	2192
Protein	1936
Ligand/ion	36
Water	220
B-factors	30.8
Protein	30.3
Ligand/ion	32.1
Water	35.7
R.m.s. deviations	
Bond lengths (Å)	0.007
Bond angles (°)	0.92
Ramachandran plot	
Most favored (%)	97.1
Allowed (%)	2.9
Outliers (%)	0

*Values in parentheses are for highest-resolution shell.

Supplementary Table 2. Primer sequences

Primer Name	Sequence
F218A Forward	gatcccattccaatatgtagcgcctgtttggggactaaagaatc
F218A Reverse	gattctttagtccccaaacaggcgctacatattggaatgggatc
R276E Forward	gcaagacatgcagtgcctgtgaggtccaaggcagaaatgctga
R276E Reverse	tcagcatttctgccttggacctcacaggcactgcatgtcttgc
F287A Forward	caaggcagaaatgctgataatatgcttgcttgtgattcctgtgatagag
F287A Reverse	ctctatcacaggaatcacaagcaagcatattatcagcatttctgccttg
D289A Forward	tgctgataatatgcttttttgtgcttcctgtgatagaggatttcata
D289A Reverse	tatgaaatcctctatcacaggaagcacaaaaaagcatattatcagca
R306E/K309E Forward	tgctgtgacccaccactttccgagatgccagaggggatgtggatttgccaagtc
R306E/K309E Reverse	gacttggcaaatccacatcccctctggcatctcggaaagtggtgggtcacagca
