# Supplementary material for: Histone H3K23-specific acetylation by MORF is coupled to H3K14 acylation
Source: Nat Commun. 2019 Oct 17;10:4724. doi: 10.1038/s41467-019-12551-5 (PMC6797804; doi:10.1038/s41467-019-12551-5)
Supplement: Supplementary file 3 — Description of Additional Supplementary Files [file 41467_2019_12551_MOESM3_ESM.pdf]

Description of Additional Supplementary Files

File Name: Supplementary Data 1
Description: Mass spectrometry analysis of MORF and MOZ complexes purified by tandem affinity chromatography presented in Fig. 1c. Spectral counts for each indicated protein are presented.

File Name: Supplementary Data 2
Description: Primers used for ChIP qPCR.

File Name: Supplementary Data 3
Description: Relative abundance of single PTMs for each analyzed cell line and the average of the four. The relative abundance of the single marks was obtained by summing the relative abundance of all the quantified peptides carrying a given PTM.

File Name: Supplementary Data 4
Description: Relative abundance of binary PTMs for each analyzed cell line and the average of the four. The relative abundance of the binary marks was calculated as for the single marks, but in this case, they were summed the relative abundances of all the peptides that contained both marks.
